# Complete Genome Sequence of *Kluyveromyces marxianus* NBRC1777, a Nonconventional Thermotolerant Yeast

**DOI:** 10.1128/genomeA.00389-15

**Published:** 2015-04-23

**Authors:** Kentaro Inokuma, Jun Ishii, Kiyotaka Y. Hara, Masao Mochizuki, Tomohisa Hasunuma, Akihiko Kondo

**Affiliations:** a Organization of Advanced Science and Technology, Kobe University, Kobe, Japan; b Department of Chemical Science and Engineering, Graduate School of Engineering, Kobe University, Kobe, Japan; c Biomass Engineering Program, RIKEN, Kanagawa, Japan

## Abstract

We determined the genome sequence of the thermotolerant yeast *Kluyveromyces marxianus* strain NBRC1777. The genome of strain NBRC1777 is composed of 4,912 open reading frames (ORFs) on 8 chromosomes, with a total size of 10,895,581 bp, including mitochondrial DNA.

## GENOME ANNOUNCEMENT

*Kluyveromyces marxianus* is a nonconventional thermotolerant yeast with status generally regarded as safe. *K. marxianus* assimilates various carbon sources, including xylose and arabinose, and is highly amenable to genetic modification (1–4). For these reasons, *K. marxianus* has attracted considerable attention as a host strain for the simultaneous saccharification and fermentation of cellulosic materials (5–7).

*K. marxianus* NBRC1777, which was isolated from Japanese soil and is available from the NITE Biological Resource Center (NBRC) in Japan, has high growth ability and ethanol productivity at elevated temperature (8, 9). To understand more about the thermotolerability of strain NBRC1777, we determined its complete genome sequence.

Sequencing of the genomic DNA of NBRC1777 was performed using an Ion Torrent Personal Genome Machine (Life Technologies, Carlsbad, CA) with 200-bp chemistry on Ion 316 chips. The sequencing reactions generated 5,060,929 reads with a mean read length of 169 bp and a total yield of 674.9 Mb (61-fold coverage of the 11-Mb genome). The raw reads were trimmed and *de novo* assembled (default settings) using CLC Genomics Workbench 7.0 (CLC bio, Aarhus, Denmark), which yielded a total of 1,644 contigs with a maximum length of 108,138 bases.

The genomic DNA of NBRC1777 was also sequenced using a PacBio *RS* II (Pacific Biosciences, Menlo Park, CA). SMRTbell libraries were prepared using a DNA template prep kit 2.0 (3 to 10 kb; Pacific Biosciences) and were sequenced on 12 silencing mediator for retinoic acid and thyroid hormone receptor (SMRT) cells (SMRT Cells 8Pac version 3; Pacific Biosciences). The raw data generated from the 12 SMRT cells consisted of 1,439,674 reads with a mean read length of 2,832 bp and a total yield of 4.06 Gb (367-fold coverage of the 11-Mb genome). The raw reads were *de novo* assembled using SMRT analysis software (version 2.1.1; Pacific Biosciences) (10) to filter subreads and circular consensus sequence reads. The assembly generated 85 contigs with a maximum length of 1,688,883 bases.

To assemble the chromosomes and mitochondrial DNA of NBRC1777, the contigs derived from the two libraries were integrated and reconstructed using CLC Genomics Workbench 7.0. The final assembly consisted of 9 contigs (8 chromosomes and mitochondrial DNA) with a total length of 10,895,581 bp and an average GC content of 40.11%. The composition of the chromosomes and the total genome size of NBRC1777 are consistent with those of *K. marxianus* DMKU3-1042 (GenBank accession numbers AP012213 to AP012221).

Annotation of the genome of NBRC1777 using BLAST analysis revealed 4,912 open reading frames (ORFs) with similar sequences to those in the nonredundant protein database from the National Center for Biotechnology Information (E-value cutoff of 10^−10^). We also identified 190 tRNAs and 6 rRNAs using the microbial genome annotation pipeline (MiGAP) (http://www.migap.org/) utilizing tRNAscan-SE 1.3 (11) and RNAmmer (12). The availability of the complete genome sequence of this strain will assist in future comparative analyses of the thermotolerability of yeast strains.

### Nucleotide sequence accession numbers. 

The genome sequences of the *K. marxianus* NBRC1777 chromosomes and mitochondrial DNA have been deposited in DDBJ/EMBL/GenBank under the accession numbers AP014599 to AP014607. The version described in this paper is the first version.
