# Erratum for Inokuma et al., Complete Genome Sequence of *Kluyveromyces marxianus* NBRC1777, a Nonconventional Thermotolerant Yeast

**DOI:** 10.1128/genomeA.00058-17

**Published:** 2017-02-02

**Authors:** Kentaro Inokuma, Jun Ishii, Kiyotaka Y. Hara, Masao Mochizuki, Tomohisa Hasunuma, Akihiko Kondo

**Affiliations:** a Organization of Advanced Science and Technology, Kobe University, Kobe, Japan; b Department of Chemical Science and Engineering, Graduate School of Engineering, Kobe University, Kobe, Japan; c Biomass Engineering Program, RIKEN, Kanagawa, Japan

## ERRATUM

Volume 3, no. 2, e00389-15, 2015. Page 1, column 1, lines 26 and 27: “silencing mediator for retinoic acid and thyroid hormone receptor” should read “single-molecule real-time.”

